# Federally mandating motorcycle helmets in the United States

**DOI:** 10.1186/s12889-016-2914-3

**Published:** 2016-03-09

**Authors:** Adam E. M. Eltorai, Chad Simon, Ariel Choi, Katie Hsia, Christopher T. Born, Alan H. Daniels

**Affiliations:** Warren Alpert Medical School, Brown University, Box G-9247, Providence, RI 02903 USA; Department of Orthopaedic Surgery, Rhode Island Hospital, Providence, RI 02903 USA

**Keywords:** Motorcycle helmet, Health policy, Injury prevention

## Abstract

**Background:**

Motorcycle helmets reduce both motorcycle-related fatalities and head injuries. Motorcycle crashes are a major public health concern which place economic stress on the U.S. healthcare system.

**Discussion:**

Although statewide universal motorcycle helmet laws effectively increase helmet use, most state helmet laws do not require every motorcycle rider to wear a helmet. Herein, we propose and outline the solution of implementing federal motorcycle helmet law, while addressing potential counterarguments.

**Conclusions:**

The decision to ride a motorcycle without a helmet has consequences that affect more than just the motorcyclist. In an effort to prevent unnecessary healthcare costs, injuries, and deaths, public health efforts to increase helmet use through education and legislation should be strongly considered. Helmet use on motorcycles fits squarely within the purview of the federal government public health and economic considerations.

## Background

Motorcycle helmets reduce both motorcycle-related fatalities and head injuries [1, 2]. Motorcycle crashes are a major public health concern which place economic stress on the healthcare system. Although statewide universal motorcycle helmet laws effectively increase helmet use [1], most state helmet laws do not require every motorcycle rider to wear a helmet.

### The human and economic cost of motorcycle crashes

In 2011, 4612 people died in motorcycle crashes in the United States, representing a 217 % increase from 1997 [3], while in 2009, there were over 90,000 motorcyclists injured [4]. Regardless, if this increase in deaths is due to the increasing numbers of motorcyclists or if it represents an increased fatality rate, the number of deaths itself is large and therefore important. Motorcycles account for less than 3 % of all registered vehicles nationwide, yet they constitute 14 % of all traffic-related fatalities [4]. Motorcyclists are 30 times more likely to die in a traffic-related crash than individuals riding in a car, for each mile traveled [4].

Helmets are beneficial in preventing head injury, as non-helmeted motorcyclists are more likely to experience traumatic brain injury (TBI) compared to those wearing helmets [5]. Median hospital costs for motorcyclists with TBI are 13 times greater than those without TBI [5], and severe TBI patients average 55 days of acute rehabilitation [6].

Injured, non-helmeted motorcyclists require substantially more healthcare resources than helmeted motorcyclists: the differential healthcare costs between non-helmeted and helmeted motorcyclist injuries account for an additional $290 million (inflation adjusted from 2006 data to represent 2015 values using Consumer Price Index data provided by the U.S. Department of Labor Bureau of Labor Statistic [7]) in healthcare costs per year [8].

Initial hospitalization and emergency treatment account for only 67 % of total medical costs in motorcycle accident victims [9]. Additional medical charges include hospital readmissions, professional fees, ambulatory care services, rehabilitation, and long-term nursing home care. Medical and productivity costs saved from helmet use are estimated to be $1,316,469.58per fatality, $186,434.37per serious injury, and $8166.06per minor injury (inflation adjusted to current year) [10]. The public must also pay for higher insurance rates, increased taxes, and lost tax revenue [11]. In a single year, the economic cost of motorcycle-related crashes total over $12.8 billion nationwide (inflation adjusted to current year) [12].

An important consideration in this major public health issue is that the cost of medical care for motorcycle crash patients is largely transferred to society. The majority of motorcycle crash victims’ medical care is paid for by public funds [13], as non-helmeted motorcyclists are more likely to be covered by government-funded health insurance or have no health insurance at all compared to helmeted motorcyclists [14].

### Helmets prevent head injuries and deaths

Helmets prevent fatalities and can reduce the number and severity of head injuries [1, 2]. After implementation of the California statewide universal motorcycle helmet law, fatalities decreased by 37.5 % [2]. Furthermore, helmets reduce the risk of head injury in motorcycle riders by 69 % [15]. In 2010 alone, an estimated 1550 motorcycle-related fatalities were prevented by helmet use and 706 more lives could have been saved if all motorcyclists had worn helmets [4].

### Helmet use increase with universal helmet laws

Universal helmet laws require all motorcyclists to wear helmets, and effectively increase helmet use [1]. Currently, each individual state determines its own helmet law. States that have enacted universal helmet laws have witnessed substantial increases in helmet use; [16–20] whereas, states that repeal universal helmet laws have witnessed substantial decreases in helmet use [16, 21–23].

### Current helmet laws

Only 20 states require all motorcyclists to wear helmets. Twenty-seven other states only require certain individuals to wear helmets when riding motorcycles, and three states have no helmet laws whatsoever [24].

### History of motorcycle helmet law in the U.S.

Motorcycles first entered mainstream markets during the 1940 and 1950s. When veterans returned from WWII, they brought home a passion for motorcycles after being introduced to them overseas [25]. In 1966, the *National Highway Safety Act* (NHSA) was passed with an eye towards decreasing motorcycle-related head injuries and fatalities. The NHSA granted states federal funds to develop programs aimed at improving traffic safety, such as vehicle registration, accident record systems, and traffic control [26]. What made this law so effective was the inclusion of a provision that allowed only those states that adopted the 1966 NHSA to be eligible to receive federal funding for highway safety programs. By 1975, every state except for California had implemented laws requiring the use of helmets while riding motorcycles [27, 28].

Opponents of the newly passed helmet statues claimed that the laws infringed upon their constitutional liberties, depriving them of their right to monitor their own safety without government intervention [29]. The American Motorcycle Association and other motorcyclist rights organization began to gain traction in court arguing that mandating motorcycle helmets represented constitutional infringement [30].

On December 13, 1975, the Senate repealed the provision of the NHSA that withheld federal funds from states unwilling to enact comparable statutes. As a result, motorcycle-related accidents increased 20 % during the next four years [31]. Motorcycle-related injuries and fatalities continued to increase over the next decade, leading to a 200 % increase in medical costs for non-helmeted motorcyclists [32–37].

Hartunian et al. compared costs between states with enforced helmet statues to those states that had repealed such laws, and found differential costs amounting to more than $412.9 million (inflation adjusted to current year) [38]. In May 1989, Senator John Chafee introduced the *National Highway Fatality and Injury Reduction Act of 1989*. The Act partly resurrected the provision attached to the 1966 NHSA by granting the federal government the power to withhold up to 10 % of federal highway aid from states refusing to enforce helmet usage. The bill was passed in 1991 with some amendments—although only 3 % of highway funds, rather than the proposed 10 %, would be withheld from states refusing to comply with the enactment [39].

In 1995, the national motorcycle lobby successfully lobbied to repeal the Federal 3 % highway safety fund penalty [31]. Arkansas and Texas were among the first states to repeal universal helmet laws in 1997. Arkansas and Texas saw fatalities rise by 21 and 31 %, respectively [40]. Even so, more states followed suit. In the coming years, Kentucky observed a 50 % increase in fatalities after the repeal of its helmet law; Louisiana observed a 100 % increase; and Florida observed a 25 % increase [31].

### Mandating motorcycle helmet usage

Convincing all motorcyclists to wear helmets will require a system-wide change. In an effort to prevent undue cost to the healthcare system due to head injury and to prevent future unnecessary deaths, Congress may wish to adopt a federal universal motorcycle helmet law. Compared to incentivizing each state to pass its own universal helmet law, a federal mandate would ensure broader adoption and expedite implementation. Although state rights are critical, motorcyclists can crash outside their home state, making this a federal issue. Given the efficacy of universal helmet laws on helmet use, a substantial impact on federal healthcare spending could be expected. Because helmets can save lives and financial resources, this solution should be attractive to a broad coalition of support from providers, insurers, and the public.

### Potential counterarguments

Special interest groups may lobby against such a federal law [41]. In the United States, certain political groups vocalize that the freedom to choose is more important than making the right choice [42]. For example, despite the increasing frequency of catastrophic mass shootings, the U.S. Congress is unwilling to pass federal gun control laws due to strong pro-gun lobbying efforts from groups that benefit from gun use [43]. To overcome vspecial interests, widespread support must be gained through voter education. For public education efforts to successfully affect behavioral change, the Organization for Economic Co-operation and Development recommends campaigns include nation-wide promotional efforts, user-friendly websites with specific information, information provided at different levels in order to best meet readers’ comprehension capabilities, use numerous available media platforms for disseminating messages, and/or relate to individual experiences by creating different approaches for unique sub-groups [44]. Voters tell their elected legislators what bills to pass. If a majority of voters feels strongly about a law, legislators will pass the law. In other words, legislators must believe that if they don’t pass such a law, they won’t be reelected. Public health initiatives are usually more successful and affect deeper change when they involve both legislation and education [44].

## Conclusions

The decision to ride a motorcycle without a helmet has consequences that affect more than just the motorcyclist. In an effort to prevent unnecessary healthcare costs, injuries, and deaths, public health efforts to increase helmet use through education and legislation should be strongly considered. Primary care providers also have opportunities to directly educate and encourage patient helmet use. Helmet use on motorcycles fits squarely within the purview of the federal government public health and economic considerations.

### Ethics

No human subjects were used in this study and was therefore not subject to Ethics or IRB review.
